# How the timing of visual feedback influences goal-directed arm movements: delays and presentation rates

**DOI:** 10.1007/s00221-023-06617-6

**Published:** 2023-04-17

**Authors:** Eli Brenner, Chris A. G. van Straaten, A. Julia de Vries, Tobias R. D. Baas, Kirsten M. Bröring, Jeroen B. J. Smeets

**Affiliations:** Department of Human Movement Sciences, Vrije Universiteit Amsterdam, Van der Boechorststraat 7, 1081BT Amsterdam, The Netherlands

**Keywords:** Frame rate, Motor control, Visually guided action, Reaching, Prehension, Contrast, Gaming, Motion-to-photon latency

## Abstract

Visual feedback normally helps guide movements to their goal. When moving one’s hand, such guidance has to deal with a sensorimotor delay of about 100 ms. When moving a cursor, it also has to deal with a delay of tens of milliseconds that arises between the hand moving the mouse and the cursor moving on the screen. Moreover, the cursor is presented at a certain rate, so only positions corresponding with the position of the mouse at certain moments are presented. How does the additional delay and the rate at which cursor positions are updated influence how well the cursor can be guided to the goal? We asked participants to move a cursor to consecutive targets as quickly as they could. They did so for various additional delays and presentation rates. It took longer for the mouse to reach the target when the additional delay was longer. It also took longer when a lower presentation rate was achieved by not presenting the cursor all the time. The fraction of the time during which the cursor was present was more important than the rate at which the cursor’s position was updated. We conclude that the way human arm movements are guided benefits from continuous access to recent visual feedback.

## Introduction

Seeing our moving limb influences how we move (Flanagan and Rao 1995; Vaidyanathan et al. 2020). This might either be because visual feedback reveals deviations from the desired trajectory (Scott 2016; Wagner and Smith 2008) or because new information is constantly used to evaluate how the movement can best proceed (in a feedforward manner; Brenner and Smeets 2022; Nashed et al. 2014; Smeets and Brenner 2004). The former is what is generally referred to as feedback control. The latter is also based on visual feedback about the moving limb, but the desired trajectory is not fixed but is planned again at each instant, giving rise to continuously updated feedforward control. In either case, one has to deal with a sensorimotor delay of at least 100 ms between visual information about the movement entering the eyes and that information influencing the movement (Brenner and Smeets 2018). When interacting with real objects, such as reaching to grasp an object with our hand, the time it takes for our movements to affect the light that enters the eyes is negligible. When we use a computer mouse to move a cursor, there is generally an additional delay (known as the motion-to-photon latency) of at least 50 ms between our actions and their visual consequences (Ng et al. 2012). This additional delay arises because the input device needs to measure a change, and both processing the input and presenting the response on the screen take time (Ng et al. 2012; Raaen and Petlund 2015). Another feature of guiding a cursor is that the visual information about one’s movement is updated intermittently rather than continuously. Do these differences make it more difficult to guide movements of a cursor than those of a hand?

We expect having an additional delay to make it more difficult to guide movements, because it increases the duration of the last part of the movement during which newly acquired information cannot be used to guide the limb (Brenner and Smeets 2018; de la Malla et al. 2018; Brenner et al. 2023). This increased difficulty will manifest itself as either a reduced precision or longer movement times (Brenner and Smeets 2011; Fitts 1954). Indeed, people are less precise when intercepting moving targets with a cursor that is displayed at the position of their hidden finger (85 Hz frame rate; 60 ms additional delay) than when they do so with the visible finger itself (de la Malla et al. 2012). Similarly, when moving their finger across a touchscreen to track a moving target with a cursor, people make larger spatial errors if there is an additional delay of 75 ms than if the additional delay is only 9 ms (Cattan et al. 2018). Moreover, increasing the additional delay by tens of milliseconds made movements noticeably slower when guiding a cursor or crosshair to a target on a screen by moving a computer mouse (Ivkovic et al. 2015; MacKenzie and Ware 1993; Spjut et al. 2019) or ‘dragging’ an item to a target on a touchscreen (Jota et al. 2013). When matching the additional delay, Spjut et al. (2019) found little influence of increasing the presentation rate from 60 to 360 Hz when moving the cursor to a target, although they did find that a low presentation rate was detrimental when the task was to keep tracking the target with the cursor. In the present study, we examine the influence of presentation rate and its relation with the additional delay in more detail.

The screen’s frame rate normally determines how often feedback provided by the cursor is updated. When a new frame appears, the cursor is shown at a position that corresponds with the mouse’s position some time earlier. It then remains at that position (or disappears) until the next frame appears. If the movement is continuously guided by where the cursor is shown, presenting the cursor for a long time at the same position (by reducing the frame rate) might be equivalent to increasing the additional delay, because the time at which the mouse reached the corresponding position gradually increases throughout the presentation at that position. It might be better to present the cursor briefly with extended time intervals during which no cursor is visible, especially if intermediate positions can be inferred from the motion (Chong et al. 2016). On the other hand, if the movement is not guided when there is no visible cursor, having extended time intervals during which no cursor is visible might be detrimental. To examine how the presentation rate and its relation with the additional delay influence performance, we varied the presentation rate in two fundamentally different ways in two separate experiments: by changing the frame rate of the monitor (Experiment 1) and by using a high frame rate of the monitor but introducing intervals with no cursor between cursor presentations (Experiment 2).

## Methods

### Equipment

We used a personal computer with a NVIDIA GeForce GTX 1050 graphical card running Ubuntu 20.04.3 LTS, combined with a 27-inch (568 by 336 mm) ASUS TUF VG279QM monitor with a resolution of 1920 by 1080 pixels. This monitor has a fast in-plane switching (IPS) panel on which images remain visible throughout each frame with little overlap between frames. We used frame rates of 60, 120 or 240 Hz in Experiment 1, and a frame rate of 240 Hz in Experiment 2. We used a ROCCAT KONE Pro Gaming mouse to measure participants’ hand movements at 1000 Hz. We determined the delay between motion of the mouse and the cursor with the help of the fast video option of a Sony RX 10^III^ camera that can record images at 1000 Hz. The camera was placed stably on a tripod.

### Delays

**Fig. 1:**
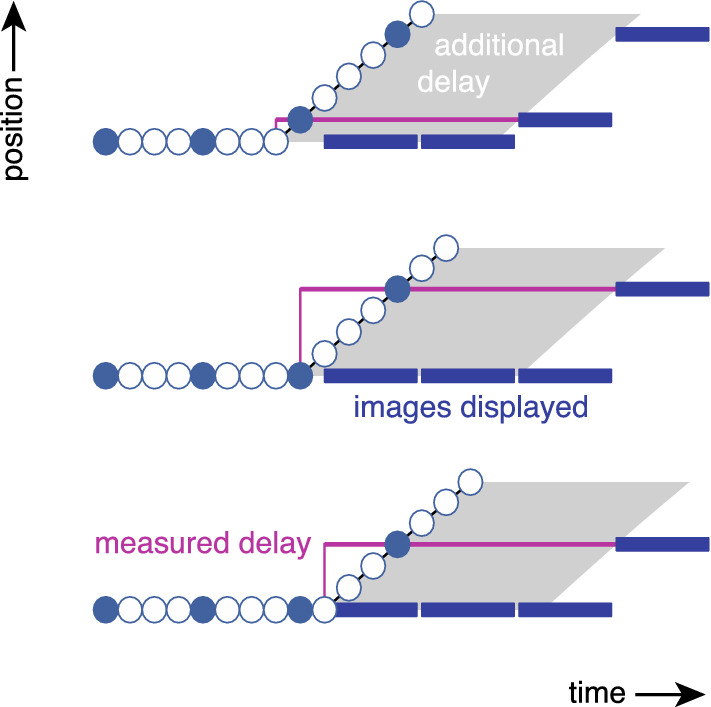
Estimating the additional delay: time between the mouse reaching a displayed position and the cursor being displayed at that position. Schematic representation of measured delays (purple horizontal lines) for three different timings of the onset of mouse movement with respect to when the cursor was presented. Circles: sampled mouse coordinates (1000 Hz; solid if presented as cursor positions; open if not). Blue rectangles: cursor presentations (in this example at 240 Hz). We used the second shortest of 25 measured delays as our estimate of the additional delay

Before testing participants, we determined the motion-to-photon latency by placing both the mouse and the cursor within view of the camera and hitting the mouse with a rigid object. We then visually inspected the images of the video recorded by the camera frame by frame, and counted the number of frames (and, therefore, of milliseconds) between the moments the mouse and the cursor moved (as in Ivkovic et al. 2015). These moments were usually easy to detect because the only displacements in the image were those of the rigid object, the mouse and the cursor. We refer to the number of frames (milliseconds) that we counted as the measured delay (Table 1). Since the moment we hit the mouse was obviously not synchronised with the frames of the monitor, and hitting the mouse at any time during the interval between two cursor presentations will give rise to a change in the cursor’s position between the presentations (Fig. 1), the counted number of frames varied across repetitions. For every condition that we wanted to test, we recorded hitting the mouse 25 times. We consider the second smallest of the 25 measured delays to be our best guess of the additional delay when continuously moving the mouse. We took the second smallest rather than the smallest in case we incidentally made an error when judging when the mouse was hit or when counting frames. The additional delay depends on the frame rate of the monitor. Unless we artificially increased the additional delay, it was 8 ms for a frame rate of 240 Hz, 10 ms for a frame rate of 120 Hz and 40 ms for a frame rate of 60 Hz. As one would expect, the range of measured delays was 4 ms at 240 Hz (delays between 8 and 12 ms), 8 ms at 120 Hz and 16 ms at 60 Hz.

**Table 1:** Brief description of some measures

Sensorimotor delay	Delay between input to the eye and resulting motor output
Additional delay	Delay between motor output and its visual consequence
Measured delay	Estimate of additional delay based on hitting the mouse once
Effective delay	Estimate of the effective delay between mouse and cursor
Presentation delay	Estimate of time taken to present a new target
Presentation rate	Rate at which new images are presented
Frame rate	Screen setting that determines the maximal presentation rate
Frame duration	1/Frame rate

In Experiment 1, we sometimes increased the additional delay by not using the cursor routines provided by the operating system to draw the cursor, but registering the position of the mouse and drawing a cursor at that position some time later using general drawing routines. We thereby obtained nine combinations of frame rate and additional delay: 8, 12, 18, 36, 59 and 95 ms additional delay at 240 Hz, 10 and 17 ms additional delay at 120 Hz, and 40 ms additional delay at 60 Hz. For each combination (condition), we determined the additional delay in the manner described above. When we increased the additional delay by drawing the cursor ourselves, the range of measured delays for the 25 repetitions was larger than when the operating system drew the cursor, because the timing of sampling the mouse coordinates and drawing the cursor is no longer optimised. In that case, we found ranges between 13 and 17 ms at 240 Hz and 15 ms at 120 Hz. We will initially ignore this increase in the range of measured delays, and will discuss how it might have influenced our findings in the discussion section.

In Experiment 2, we always used the shortest possible additional delay (8 ms at 240 Hz), but we did not always present the cursor on each frame. We either presented it on each frame, or skipped 1, 2, 3, 4, 5, 7, 9 or 11 frames after each presentation, so that the cursor was visible on all frames or only every second, third, fourth, fifth, sixth, eighth, tenth or twelfth frame (nine conditions). Note that we only skipped cursor presentations; the target remained visible. As reducing the time that the cursor was visible reduced its effective contrast, we also included three conditions in which we presented the cursor at 240 Hz with contrasts that matched the time-averaged contrast when skipping selected numbers of frames. Experiment 2 therefore had 12 conditions. To match skipping 3, 7 or 11 frames, we presented the cursor at a contrast of a quarter, an eighth or a twelfth of the original contrast. To do so, we calibrated the luminance of the screen with a Minolta CS 100A Chroma Metre.

**Fig. 2:**
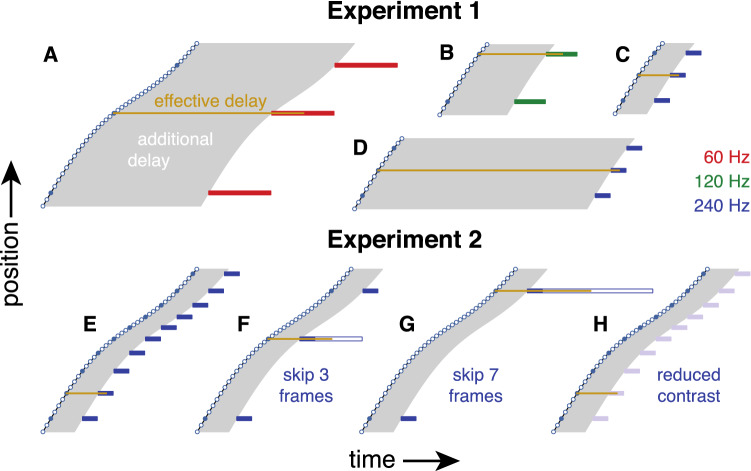
Schematic representation of the *effective delay* (length of golden line). In Experiment 1, we varied the effective delay by changing the frame rate (**A**: 60 Hz; **B**: 120 Hz; **C**, **D**: 240 Hz) and by either presenting the cursor with the shortest possible additional delay (**A**: 40 ms; **C**: 8 ms) or by intentionally increasing the additional delay (**B**: 17 ms; **D**: 59 ms). In Experiment 2, the frame rate was always 240 Hz, but we varied the presentation rate and thereby the effective delay by sometimes skipping frames. Skipping no frames (**E**) was the same as having the shortest additional delay at 240 Hz in Experiment 1 (**C**). Skipping 3 (**F**) or 7 (**G**) frames meant that no cursor was shown for 12 or 28 ms (open rectangles) between consecutive 4 ms cursor presentations. We also varied the contrast of the cursor without skipping frames (**H**). For an explanation of the symbols, see Fig. 1

The additional delay that we considered in the previous paragraphs is the time until a new cursor position is shown. If participants continuously guide their movements on the basis of the latest shown cursor position, the movements will be guided by a new cursor position from this additional delay until the next cursor position is presented. For a lower frame rate, and hence a longer frame duration, the same position will therefore be considered for a longer time, so the *effective delay* will be longer. We define the *effective delay* as the time between the mouse reaching a presented position and the moment half way between when the cursor was first presented at the corresponding position on the screen and when it was first presented at a different position (Fig. 2). In Experiment 1, the *effective delays* were therefore 10, 14, 20, 38, 61 and 97 ms at 240 Hz, 14 and 21 ms at 120 Hz, and 48 ms at 60 Hz. In Experiment 2, the *effective delay* was 10 ms in the conditions in which no frames were skipped, and 12, 14, 16, 18, 20, 24, 29 and 33 ms in the conditions with skipped frames.

When analysing the data, a final delay to consider is the delay in presenting a new target. This *presentation delay* is the time it takes to show a new target from the moment that the progress of the experiment indicates that a new target should be presented. In terms of Fig. 1, it is, therefore, equivalent to the time from when the mouse starts to move until the first frame with cursor motion, which is slightly longer than the time from when the cursor reached the position at which it was shown until the first frame with cursor motion (the additional delay). Since we drew the target as quickly as possible, the best estimate of this delay is the average of the measured delays when not letting the system draw the cursor but registering the position of the mouse and drawing a cursor at that position as quickly as possible. This estimate of the presentation delay is 17 ms for a frame rate of 240 Hz, 22 ms for a frame rate of 120 Hz, and 58 ms for a frame rate of 60 Hz.

### Participants

There were 20 participants in the first experiment (11 male, 9 female; on average 27 years old; range 18–65 years) and 20 other participants in the second (11 male, 9 female; on average 20 years old; range 18–31 years). Since the study was not designed to examine whether we could statistically reject a specific hypothesis, no power analysis was performed. We selected people who normally used their right hand to move the mouse, because the mouse that we used was designed for use with the right hand. All participants volunteered to take part in the experiment after being informed about what they would be required to do and signing a consent form (in accordance with our ethical approval). The participants did not know precisely what we were studying, but in some cases, they could guess what we were manipulating: the longest additional delay was noticeable, the cursor flickered visibly at low presentation rates, and the contrast looked lower when either the presentation rate or the contrast itself was reduced. Participants were informed that they would perform several blocks of movements that would last either 2 min (Experiment 1) or 1 min (Experiment 2). Their task was to move the cursor to as many targets as possible within that time. The participant could adjust the position of the screen, mouse and chair, as well as the height of the screen and chair, to perform the task comfortably. At the distances that they chose, 1 cm on the screen corresponds to approximately 1° of visual angle.

### General procedure

**Fig. 3:**
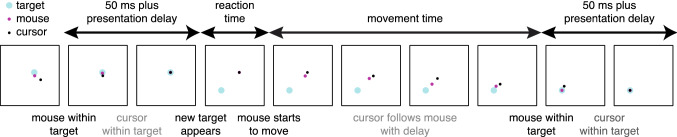
Schematic representation of the task (not to scale). The position in the figure that corresponds with the mouse position (purple disc) was obviously not visible on the screen, but is shown here to clarify the flow of events. The critical moments are indicated in black text; other events are mentioned in grey text

The participants’ task was to move a 0.7 cm diameter black disc (the cursor) to a 1.3 cm diameter blue disc (the target) on a white background. The flow of events was determined by the mouse’s movements (Fig. 3), so the timing of events relative to how the cursor moved differed between conditions. The participant had to move the mouse such that its position corresponded with a cursor position on the screen that was within the target. If the mouse remained within the target for 50 ms, the moment at which it entered the target was considered to be the end of the movement. If the mouse left the target before the 50 ms had expired, it had to move back into the target and remain there for 50 ms from when it re-entered the target. After the 50 ms and the presentation delay the target disappeared a new target appeared at a distance of 11.8 cm from the original target in a random direction (selected from all directions for which the whole target would be visible on the screen). The block did not terminate as soon as its duration expired, but as soon as a new target would be shown after its duration expired.

### Procedure of Experiment 1

Participants started with a practice block of movements with an effective delay of 38 ms and a frame rate of 240 Hz. They then performed separate blocks of movements with effective delays of 10, 14, 20, 38, 61 and 97 ms at a frame rate of 240 Hz, 14 and 21 ms at a frame rate of 120 Hz, and 48 ms at a frame rate of 60 Hz. These nine blocks of movements were presented in random order. Participants could rest as long as they liked between the 2-min blocks of movements.

### Procedure of Experiment 2

Participant started with a practice block of up to 2 min with an effective delay of 10 ms (black cursor presented at 240 Hz). If they indicated that they had practised enough, the practice block was terminated before the end of the two minutes. They then performed separate blocks of movements with a black cursor shown on every frame or every 2nd, 3rd, 4th, 5th, 6th, 8th, 10th or 12th frame. In three additional blocks, the cursor had one of three grey levels (chosen so they had 1/4, 1/8 or 1/12 of the contrast of the black cursor) and was shown on every frame. The 12 blocks of movements were presented in random order. Participants could rest as long as they liked between the 1-min blocks of movements.

### Analysis

Our main measure was the movement time: the time between movement onset and the end of the movement. Movement onset was defined as the moment the mouse coordinates left the region corresponding with the previous target. The end of the movement was defined as the moment the mouse coordinates entered the region corresponding with the current target to remain within that region for 50 ms (Fig. 3). To further characterise the participants’ behaviour we also determined the reaction time. The reaction time was the time between our estimate of when the target appeared (see final paragraph of “Delays” section) and movement onset. Defining movement onset as the moment the mouse coordinates left the region corresponding with the previous target meant that the reaction time includes the first part of the movement.

For each block, we characterised the movement time and the reaction time by their median values. We used median values to avoid excessive influences of occasional outliers, such as movements that overshot the target and therefore, lasted exceptionally long because they had to return to bring the cursor within the target. To quantify the time lost in this manner we determined an *overshoot time*: the time between when the mouse coordinates first entered the region corresponding with the current target and the end of the movement. This overshoot time was zero if the mouse entered the target and stayed there, but it was larger than zero if the mouse did not remain within the target for at least 50 ms when it first reached the target. In this case, we obviously used the mean value because the median value is zero. We also determined the fraction of trials in which participants overshot the target.

To further evaluate changes in participants’ movements, we determined velocity profiles. To obtain a velocity profile that we could average across movements, we split the mouse’s path from movement onset until the first moment it entered the target into 50 equal distances, and determined the velocity when the mouse was at each of the 51 positions defined by those distances. The velocity at each of those positions was the combined lateral and sagittal velocity of the mouse, each of which was determined by simultaneous differentiation and smoothing with a Savitzky–Golay filter. We applied this filter to each sampled position of the mouse by fitting a second order polynomial to all points within 20 ms of the moment the mouse reached the position in question. We took the fit value for the first order parameter as the mouse’s velocity at that moment.

The five measures (median movement time, median reaction time, mean overshoot time, fraction of trials with an overshoot, and mean velocity profile) were determined separately for each block of each participant. They were then averaged across participants for blocks of the same *condition* (blocks with the same effective delay, frame rate, number of skipped frames and cursor contrast). We were interested in systematic differences across conditions, irrespective of any overall differences in performance across participants. We, therefore, normalised the movement times, reaction times and overshoot times before determining the confidence interval. We divided all of each participant’s values for the measure in question by that participant’s mean value across all conditions. For each condition, we then determined the mean and 95% confidence interval of these scaled values across participants. Finally, we multiplied all the mean values and confidence intervals by the overall mean for that measure. We will refer to the confidence interval that we obtained in this manner as the normalised 95% confidence interval.

## Results

### Experiment 1

**Fig. 4:**
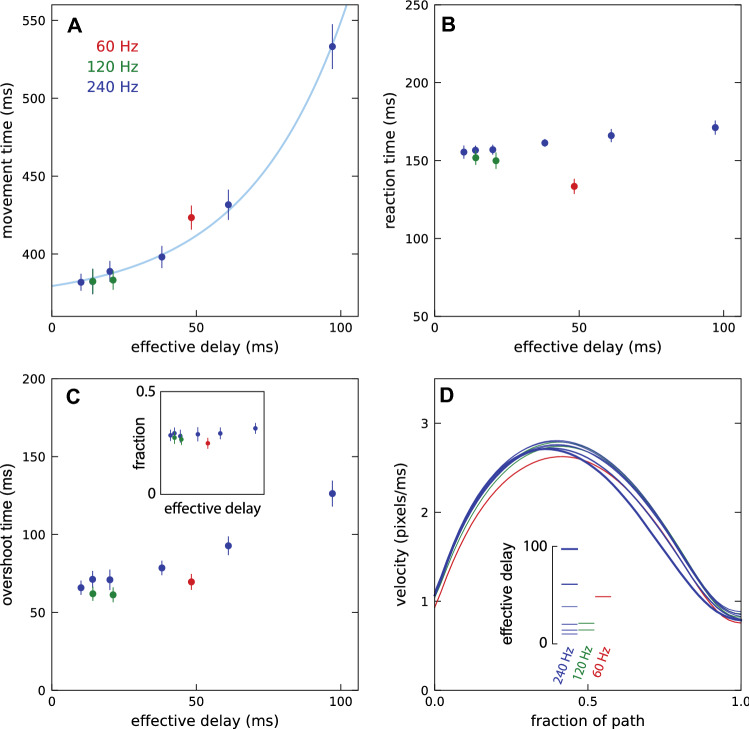
Results of Experiment 1. Colours indicate the frame rate. Error bars are normalised 95% confidence intervals. **A** Movement time. The curve is an exponential function of the form $MT=a+b\times {e}^{c \, \mathrm{delay}}$ that we fit to the 240 Hz data. A blue point is hidden behind the green one for an effective delay of 14 ms. **B** Reaction time. **C** Overshoot time. The inset shows the fraction of trials with overshoots. **D** The velocity as a function of position. The thickness of the curves indicates the effective delay: thicker curves for longer delays. The velocity does not start and end at zero because the path is taken from when the mouse leaves the previous target until it first enters the new one, ignoring the initial and final movement within the target, and the cursor possibly overshooting the target so that the first time it enters the target is not the end of the movement

As expected, the movement time increased with the effective delay (Fig. 4A). In accordance with the reasoning behind our definition of the effective delay, for the same effective delay the movement time was not longer for a 120 Hz frame rate (green symbols) than for a 240 Hz frame rate (blue symbols). However, it was slightly longer for a 60 Hz frame rate (red symbol). Thus, the effective delay may not be the only relevant factor: the presentation rate may also be important. We will return to this in Experiment 2 in which we manipulate the presentation rate independently of the frame rate.

For the 240 Hz conditions, the reaction time also increased with an increasing effective delay (blue symbols in Fig. 4B), but this increase was much more modest than the increase in movement time. Surprisingly, the reaction time was shorter for lower frame rates (red and green symbols). The overshoot time also increased with increasing effective delay and was also shorter for lower frame rates at an equivalent effective delay (Fig. 4C). Combining the clear increase in the overshoot time with the very modest increase in the fraction of trials with an overshoot (inset of Fig. 4C) means that each overshoot cost more time when the effective delay was longer, which makes sense because it takes longer for the overshoot to be detected since the cursor is delayed. The velocity was lower throughout the movement when the frame rate was 60 Hz (red curve in Fig. 4D). It was lower during the second half of the movement for the two longest effective delays (61 and 97 ms; two thickest blue curves in Fig. 4D).

### Experiment 2

**Fig. 5:**
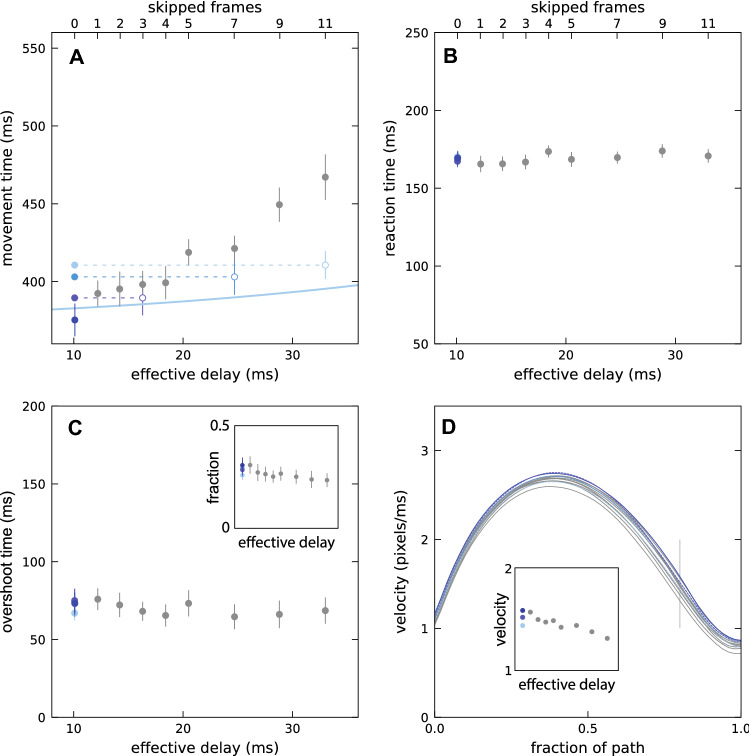
Results of Experiment 2. Symbols are blue if the cursor presentation rate is the same as the frame rate (240 Hz) and grey if the cursor presentation rate is lower than the frame rate. Brighter shades of blue indicate lower contrast of the cursor. The horizontal positions of the grey symbols (effective delays) depend on the presentation rate (number of skipped frames). Error bars are normalised 95% confidence intervals. **A** Movement time. Filled symbols indicate the actual combination of effective delay and movement time. Open symbols reproduce the movement times when the cursor had a reduced contrast at the horizontal positions of the conditions with skipped frames that have the same time-averaged contrast. The blue curve is the fit from Fig. 4 (it looks shallower because of the smaller range of effective delays). **B** Reaction time. **C** Overshoot time. The inset shows the fraction of trials with overshoots. **D** Velocity profiles. Colour coding of the curves correspond to that of the symbols in the other panels. Thicker curves indicate longer effective delays. Dashes indicate a reduced contrast. The velocity at 80% of the path (vertical grey line) is shown more clearly in the inset (the blue points representing the two intermediate contrasts are at the same position, so one is hidden)

In Experiment 2, the frame rate of the monitor was always 240 Hz, but the cursor’s presentation rate varied between conditions. This presentation rate is the rate at which frames were presented in which the cursor was shown (for 4 ms). Decreasing the rate at which cursor positions were presented increased the movement time by more than one would expect on the basis of the effective delay (grey symbols above curve in Fig. 5A). Even only skipping one frame (resulting in a presentation rate of 120 Hz) is detrimental (leftmost grey dot above darkest blue dot and above curve). Decreasing the contrast without changing the presentation rate also increased the movement time (lighter blue dots), but the increase was modest compared to the condition in which the same time-averaged contrast was achieved by skipping frames (open symbols below grey ones).

Skipping frames or changing the cursor’s contrast did not clearly influence the reaction time (Fig. 5B) or the overshoot time or fraction of trials with overshoots (Fig. 5C). Skipping more frames gave rise to lower velocities throughout the movement (lower, thicker curves in Fig. 5D), but the differences were particularly evident near the end of the movement, when the velocity was low so that its influence on the movement time was relatively large.

## Discussion

It took longer to move to targets when feedback about the movement was delayed (Fig. 4A), confirming earlier findings with a variety of similar tasks (Ivkovic et al. 2015; Jota et al. 2013; MacKenzie and Ware 1993; Spjut et al. 2019). In Experiment 1, we established the relationship between what we defined as the *effective delay* (see “Delays” section of the methods) and the time it took to move to the target. This relationship is shown by the curve in Fig. 4A. We subsequently used this relationship to evaluate how the presentation rate influenced performance, independently of its influence on the effective delay. In the first experiment, we changed the presentation rate by changing the screen’s frame rate. There was an increase in the time taken to reach the target for a frame rate of 60 Hz with respect to the time taken at 240 Hz, but no increase in the time taken at 120 Hz. In Experiment 2, we did not vary the frame rate, but we varied the cursor’s presentation rate by not presenting the cursor on all frames. We found that the time taken to reach the target increased faster with the number of skipped frames than predicted by the change in effective delay. It also increased faster than predicted by the reduction in the time-averaged contrast. Thus, the presentation rate matters when the cursor is not presented on all frames, but it does not always matter when the monitor’s frame rate is changed. How can we explain this?

The simplest explanation is that it is not really the presentation rate that matters, but the fraction of the time during which a cursor is visible. That would explain why reducing the screen’s frame rate does not always influence performance, but not presenting the cursor on all frames does. The only finding that is inconsistent with this explanation is the longer movement time than would match the effective delay at a frame rate of 60 Hz (red point above curve in Fig. 4A). We, therefore, take a closer look at this finding before further considering why the fraction of the time during which a cursor is visible might matter.

### The 60 Hz frame rate

Completion times for a somewhat similar task have previously been reported not to depend on the frame rate (Spjut et al. 2019). In that study, they tested frame rates from 60 to 360 Hz. This is less inconsistent with our findings than it might seem, because the sum of the reaction time and the movement time is not longer at 60 Hz in our study either. But why was the reaction time particularly short for the 60 Hz frame rate in our study (red point in Fig. 4B)?

**Fig. 6:**
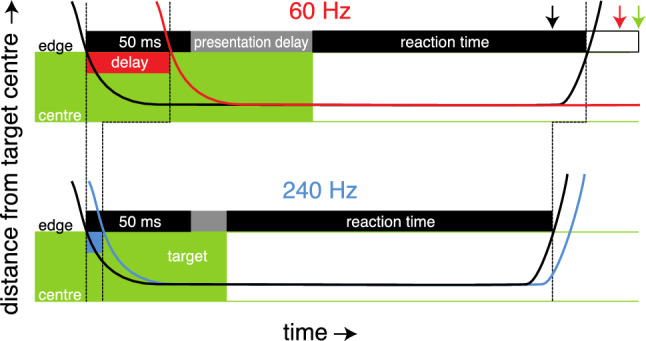
Schematic representation, more or less to scale, of the timing of the reaction time for the shortest delays at 60 Hz and 240 Hz frame rates. Green areas indicate the presence and radius of the target at which the original movement ended. The black curves indicate the distance of the mouse from that target’s centre as a function of time. The red and blue curves show the corresponding cursor distances from the target centre (same curves shifted by the additional delay: width of the red and blue rectangle). The green arrow indicates when the mouse coordinates would leave the region corresponding with the target that has just disappeared if the time from when the new target appeared (the reaction time) were the same at 60 Hz as at 240 Hz. The back and red arrows indicate when this would happen if the time from when the mouse and cursor had entered the target were the same at 60 Hz as at 240 Hz (same distance from first and second dashed line)

We propose that the reaction time is influenced by how long it takes to present a new target from when the mouse reaches the original target (Fig. 6). The reaction time is defined with respect to when the new target appears, but participants might not only be influenced by when the new target appears. They may also have a tendency to start moving a certain time after the mouse or cursor reach the original target. A tendency to start moving a certain time after the mouse reached the target could account for the shorter reaction time for the 60 Hz frame rate than for the 240 Hz frame rate, because the movement started longer after the mouse reached the target (after the black arrow in Fig. 6) despite starting sooner after the target appeared (before the green arrow in Fig. 6).

There might also be a modest tendency to start moving a certain time after the cursor reached the target, possibly due to it taking longer to check that the movement was successful when the cursor is delayed, because we also see a slight increase in reaction time with the effective delay for the 240 Hz frame rate. In accordance with the proposal that the reaction time depends on the timing of the presentation of new targets, we do not see such differences in reaction time across conditions in Experiment 2, where new targets were displayed at fixed times with respect to when the mouse reached the target. Therefore, the longer movement time for the 60 Hz frame rate in our study is probably the result of when new targets appeared, rather than of the presentation rate.

### Time during which a cursor is visible

The strongest support for the fraction of time during which a cursor is visible being critical is that presenting the cursor at 120 Hz by doubling the frame duration did not increase the movement time (two green points in Fig. 4A), whereas presenting the cursor at 120 Hz by only presenting it every second frame at 240 Hz did (leftmost grey point above the darkest blue point in Fig. 5A). The main difference between these two ways of achieving a 120 Hz presentation rate is that the cursor remains visible for 8 ms in the former, but it is only visible for 4 ms in the latter. At such high presentation rates, one does not notice that the cursor is absent half the time, but when it is only visible half the time one could notice that it has a lower time-averaged contrast. Reducing the contrast without skipping frames also increased the movement time (light blue symbols in Fig. 5A), but skipping frames increased the movement time much more than a comparable time-averaged reduction in contrast (grey symbols above open symbols in Fig. 5A). Thus, the reduced time-averaged contrast presumably cannot fully account for the difference between reducing the frame rate to 120 Hz and only presenting the cursor on half the frames at a frame rate of 240 Hz. But why does a reduction in contrast influence the movement time at all?

Reducing the contrast of the *target* of a goal-directed movement increases the latency of adjustments to displacements of such a target (Veerman et al. 2008). Presumably, reducing the contrast increases the time it takes to process visual information within the eye and brain, and thereby the sensorimotor delay. In terms of guiding the movement, increasing the sensorimotor delay by reducing the contrast is presumably equivalent to introducing an additional delay between the movements of the mouse and cursor. It is unlikely to matter whether visual information about the moving hand takes longer to process because the contrast is low, so processing within the eye and brain is slower, or because information about the hand movement is delayed before entering the eye. If this reasoning is correct, we can estimate from the data that reducing the contrast to 1/4 of the original value increases the sensorimotor delay by about 10 ms, while reducing it to 1/12 of the original value increases it by about 40 ms (horizontal distance between the solid blue points and the curve in Fig. 5A; for the lower contrast the curve has to be extrapolated beyond the panel).

The idea of comparing performance for the same time-averaged contrast arose from the notion that for brief presentations only the total amount of light matters (Bloch 1885), which is consistent with the cursor looking the same with skipped frames and with a lower contrast, at least for high presentation rates when it is static. However, even for static stimuli, this only holds under specific conditions (Barlow 1958; Greene 2013) for some judgments (Scharnowski et al. 2007). For a moving cursor, it is more complicated, because both the number of positions that are shown and the contrast at each position change. Our results show that the benefit of having more positions outweighs the cost of having a reduced contrast at each position. Since we know that it is not really the number of positions that matters, because having more positions did not improve performance despite the time-averaged contrast remaining the same when the frame rate was increased from 120 to 240 Hz in Experiment 1 (or when it was increased from 60 to 360 Hz in Spjut et al. 2019), we can conclude that the time with no visible cursor is detrimental.

When the cursor was only visible about 8% of the time (for 4 ms once every 50 ms, so that the cursor was presented 9 or 10 times during the whole movement) the increase in the movement time was equivalent to having an effective delay of about 80 ms (the horizontal position on the curve in Fig. 4A with the same movement time). This is 47 ms longer than the calculated effective delay of 33 ms for the presentation rate of 20 Hz (skipping 11 frames between cursor presentations). However, not seeing the cursor for part of the time is not simply equivalent to having a longer delay, because we do not see equivalent changes in overshoot time or in the velocity profile.

### Limitations and remaining questions

Our conclusions are mainly based on comparing performance at 240 Hz and 120 Hz presentation rates. The planned comparison with a 60 Hz presentation rate is messed up by the change in reaction time. As already mentioned, the results show that the time it took to react to new targets appearing depends on the time at which the target appears (with respect to when the former movement ended). In retrospect, it would have been better to always have the target appear at a fixed time after the end of the previous trial, which would mean delaying the appearance of new targets for the higher frame rates in Experiment 1. It remains to be seen whether this is enough to equate reaction times, or whether the target has to appear slightly earlier for longer delays to compensate for an influence of waiting for the cursor to reach the target (see Fig. 6).

As mentioned in the Methods section, the range of measured delays was larger when we used general drawing routines to draw the cursor (in order to increase the additional delay in Experiment 1) than when we let the operating system draw the cursor. This is understandable, but it means that we underestimated the effective delay in all the cases in which we artificially increased the delay, because we defined the effective delay using the frame duration. Correcting for the larger range is not straightforward, because in contrast to the measured values for when the operating system drew the cursor, for which the values were evenly distributed across the range, when general drawing routines were used to draw the cursor the distribution was skewed (the median value was systematically smaller than the mean value). Thus, we do not know exactly by how much we underestimated the effective delay, but we do know that all the points except the red one and the leftmost green and blue ones in Fig. 4A–C should be shifted slightly to the right. This shift is presumably slightly less than half the difference between the measured range and the frame duration, so almost 5 ms. It is evident that shifting the mentioned points about 5 ms to the right would not change our conclusions.

We had expected delays to make participants overshoot the target, because a long delay makes the cursor appear to have moved less far than it actually had, to which we expected participants to respond by moving the mouse farther. The fraction of trials in which the cursor overshot the target might be slightly larger for longer delays, but participants did not overshoot the target much more often when there was a long delay (insets in Figs. 4C, 5C). In contrast, the *overshoot time* did clearly increase with the delay (at least in Experiment 1; Fig. 4C). Presumably, a longer delay increased the time it took to realise that one had overshot the target, because such realisation is based on movements of the cursor rather than of the mouse. The stronger asymmetry in the velocity profile for the longest delays (thick blue lines in Fig. 4D), with a particularly low velocity near the end of the movement, might be a response to this increased cost of overshooting the target in terms of the time lost every time one does so: participants did not overshoot the target more with a long delay, as we had expected, because they compensated for the increased cost of overshooting the target by moving more slowly, especially as they approached the target.

If the cursor being visible more of the time is more important than the number of cursor positions that are shown, we expect performance to be better on modern gaming screens on which the cursor is constantly visible than on old CRT screens on which the cursor is flashed briefly on every frame. MacKenzie and Ware (1993) used a CRT screen at 60 Hz, and report a movement time of about 800 ms for movements of a comparable difficulty to ours (ID 3.6) for their shortest delay (8 ms). This is indeed clearly poorer performance than we found at 60 Hz and a longer delay in Experiment 1, or when presenting the cursor quarter of the time with a similar delay in Experiment 2. However, there are many differences between our study and theirs, so we cannot confidently attribute the difference in performance to the duration for which the cursor was visible.

Ivkovic et al. (2015) found considerably longer times than ours for a similar task and index of difficulty using a gaming monitor at 120 Hz. The reason might be that their participants had to press a button once they reached the target. Having to press a button when one reaches the target presumably takes additional time, especially considering that we measured time until the mouse entered the target rather than until it reached a standstill within the target. Having to press a button might also increase the influence of delays with respect to our method, because participants might wait until the cursor is within the target before pressing the button, which obviously increases the time by the additional delay. That continuous availability of updated visual information is important for guiding movements is supported by the finding that saccade endpoints are more precise when *targets* are visible more of the time, even when the time-averaged contrast is equated (Goettker et al. 2020).

## Conclusion

Our most important observation is that movements are guided more efficiently when the cursor is visible all the time than when it is only visible part of the time, even when the rate at which new positions are presented is high. The cursor being visible more of the time even seems to be more important than the number of cursor positions that are shown. This is consistent with the idea that people continuously use visual information to guide their movements (Brenner & Smeets 2018; Brenner et al. 2023). In the current experiments, the task was to move a cursor to a target by moving one’s hand in a different plane, so participants had to rely on visual information about the cursor as well as the target. When the task is to move one’s hand to a target one can rely more on haptic and efferent information about the hand. Although this might decrease the importance of having continuous visual feedback about the position of the hand, it would not question the continuous nature of the way movements of the hand are controlled. Having continuous visual feedback about one’s movement is, therefore, presumably always beneficial. This even holds for presentation rates for which one cannot detect whether visual feedback is always present (60 Hz and higher).

## Data Availability

The programmes and raw data can be found at https://osf.io/y4g9h/?view_only=56e15eaf6bd547519dd58f158cef329b.
